# Widespread Deposition in a Coastal Bay Following Three Major 2017 Hurricanes (Irma, Jose, and Maria)

**DOI:** 10.1038/s41598-019-43062-4

**Published:** 2019-05-08

**Authors:** Trevor N. Browning, Derek E. Sawyer, Gregg R. Brooks, Rebekka A. Larson, Carlos E. Ramos-Scharrón, Miguel Canals-Silander

**Affiliations:** 1 School of Earth Sciences, The Ohio State University, 125 S. Oval Mall, Columbus, Ohio 43210 USA; 2 Department of Marine Science, Eckerd College, 4200 54th Ave. S., St. Petersburg, Florida 33711 USA; 3 Department of Geography & the Environment, University of Texas at Austin, 305 E. 23rd Street, Austin, Texas 78712 USA; 4 UPRM Center for Applied Ocean Science and Engineering, Department of Engineering Sciences and Materials, University of Puerto Rico at Mayaguez, PR-108, Mayaguez, 00682 Puerto Rico

**Keywords:** Physical oceanography, Sedimentology, Natural hazards, Environmental impact

## Abstract

In 2017, three major hurricanes (Irma, Jose, and Maria) impacted the Northeastern Caribbean within a 2-week span. Hurricane waves can cause physical damage to coastal ecosystems, re-suspend and transport antecedent seafloor sediment, while the associated intense rainfall can yield large influxes of land-derived sediment to the coast (e.g. burial of ecosystems). To understand sedimentation provenance (terrestrial or marine) and changes induced by the hurricanes, we collected bathymetry surveys and sediment samples of Coral Bay, St. John, US Virgin Islands in August 2017, (pre-storms) and repeated it in November 2017 (post-storms). Comparison reveals morphologic seafloor changes and widespread aggradation with an average of ~25 cm of sediment deposited over a 1.28 km^2^ benthic zone. Despite an annual amount of precipitation between surveys, sediment yield modeling suggests watersheds contributed <0.2% of the total depositional volume. Considering locally established accumulation rates, this multi-hurricane event equates to ~1–3 centuries of deposition. Critical benthic communities (corals, seagrasses) can be partially or fully buried by deposits of this thickness and previous studies demonstrate that prolonged burial of similar organisms often leads to mortality. This study illuminates how storm events can result in major sediment deposition, which can significantly impact seafloor morphology and composition and benthic ecosystems.

## Introduction

**Figure 1:**
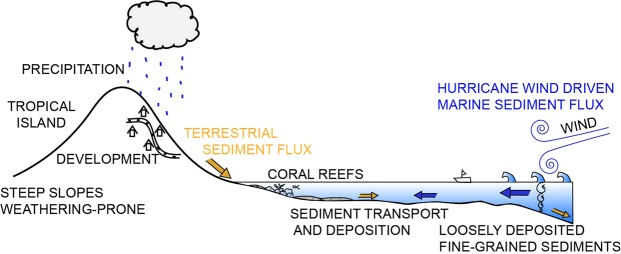
A conceptualized schematic of terrestrial and marine sediment transport in the tropical coastal system characterized by two sediment transport regimes: one moving marine-derived sediment via hurricane waves and the other terrigenous sediment from the watershed via rainfall.

Tropical cyclones are among the most destructive natural hazards on Earth^1^. Approximately 133 tropical cyclones form annually in the world’s oceans (86 tropical storms and 47 hurricanes, on average over the past 30 years)^2^. Of those, approximately half impact Southeast Asia (37%) and the Caribbean (14%)^2^. Both of these regions are composed primarily of open water with many small tropical islands with infrastructures easily damaged and/or destroyed by tropical cyclones^3^. Tropical islands have been developing rapidly for decades due to growing populations and economies^4^. The land-use changes that accompany population growth often enhance land-derived sedimentation, which can negatively affect coral reefs and other nearshore ecosystems^5,6^ (Fig. 1). Large waves from storms can also transport marine-derived sediment onshore and into the coastal zone^7^ (Fig. 1). The degradation of coastal ecosystems, exacerbated by sedimentation among other stressors^8^, threatens ecosystems, reef fish populations, and consequently the economies of tropical nations^9–12^.

Coastal ecosystems are critical to economic, cultural, and ecologic health^12–14^, yet are vulnerable to hurricanes^15^. For example, calcareous algae are reef builders^16^, seagrasses serve as fish nurseries^17,18^, and coral reef communities create diverse habitats^14^ and buffer the coast from wave action and storms^19^. Coastal vegetation such as seagrass communities are an important source for carbon capture and storage and do so ~2x more efficiently than tropical rainforests^20^. Coastal marine communities are resilient to small storm events^21,22^ in which the circulation of water and sediment can actually stimulate diversity^8^, cover^23^ and new growth^24^ while having little positive or negative effect on other communities^25^. Conversely, large storm events can result in full mortality due to burial of most coral species^5,26^, some calcareous algaes^27^ and some tropical seagrasses^28–31^ (exacerbated by accompanying salinity decreases^32^). If uncovered rapidly enough, seagrasses^31,33^, calcareous algaes^25^ and some corals^8,34,35^ are able to partially recover. Additionally, breakage and fragmentation accompanying burial during hurricanes is not necessarily lethal for some coral communities^35,36^ and fragmentation can be inconsistent and patchy^37^. Broken calcareous algae plates are often found following large storms^38^ (including in this study) despite their resilience and common recovery^25^. Specifically in the Caribbean, hurricanes have led to dramatic declines in dominant coral species^39–41^.

**Figure 2:**
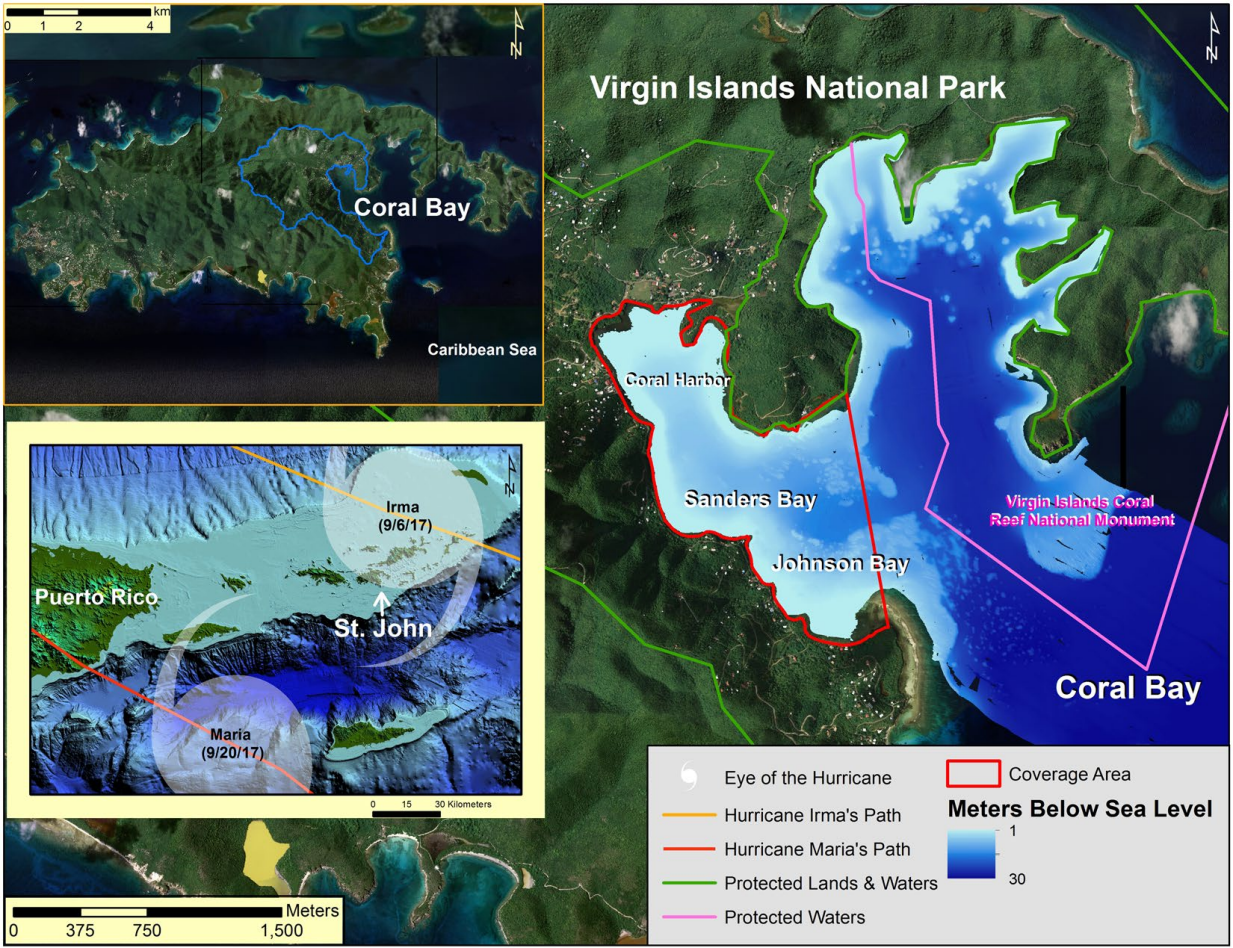
Bottom Left: Eastern Puerto Rico and the US and British Virgin Islands with the 2017 hurricane tracks from Category 5 Hurricanes Irma and Maria^52,55^. St. John, US Virgin Islands sits on a shelf ~60 meters below sea level (This map was generated using NOAA’s U.S. Virgin Islands 1 arc-second MHW Coastal Digital Elevation Model^96^ with ESRI ArcGIS 10.6, www.esri.com). Top Left: St. John, US Virgin Islands showing the watershed source area (This map and the map shown to the right were generated using US Army Corps of Engineers US Virgin Islands Orthophoto Mosaic^97^ using ESRI ArcGIS 10.6, www.esri.com). Right: Coral Bay, St. John with its protected waters and lands. Bathymetry shown was collected after the 2017 hurricane season in November and December of 2017.

St. John, US Virgin Islands (USVI) is a volcanic island in the Caribbean Leeward Islands (Fig. 2)^42^. The coastal system has two dominant sources of sediment: marine-derived carbonates and terrigenous-derived volcaniclastics^43,44^. The steep slopes, short pathways to the coast, and weathered volcanic soils of the island make it particularly susceptible to land-based sediment erosion and coastal deposition from both human and natural causes^45–47^ (Fig. 1). There are no perennial rivers on St. John, only a series of ephemeral gullies activated by extreme rainfall events^48–51^. This allows a situation in which the land-based sediment transport system is either turned ‘on’ (during rainfall events) or ‘off’ (Fig. 1). These characteristics create an easily distinguishable terrestrial vs. marine signal in the geologic record following any erosional and depositional event (Fig. 1).

The year 2017 was historic, as numerous major hurricanes severely impacted many Caribbean Islands and portions of the continental United States. Hurricane Irma was one of the longest and most intense hurricanes on record and the strongest storm (maximum sustained wind speed = 83 m s^−1^) to make landfall in the Leeward Islands^52,53^. In terms of accumulated cyclone energy, Hurricane Irma alone equaled an entire average Atlantic hurricane season^52^. Irma made landfall at near peak intensity on Virgin Gorda, British Virgin Islands on September 6^th^, 2017, ~36 km northeast of Coral Bay, St. John, USVI^52^ (Fig. 2, Table 1). As the storm moved in the west-north-west direction, the eyewall passed over Coral Bay. The eyewall of the hurricane often has the greatest wind speed and heaviest rainfall of the storm cell^2^ (Table 1). Thus, Irma resulted in widespread damage throughout St. John.

**Table 1:** Hurricane statistics for 2017 Atlantic Category 5 Hurricanes Irma and Maria and Category 4 Hurricane Jose.

Location Recorded	Date/time (UTC)	Distance from Coverage Area (km)	Barometric Pressure (mb)	Wind Speed (m/s)	Rainfall (mm)	Wave Height (m)	Wave Period (s)
***Hurricane Irma***							
Peak Intensity E of St. John (ocean)^a^	9/6/17 11:15	172.0	914	79.7	N/A	N/A	N/A
S of St. John (ocean)^b^	9/6/17 13:00	11.0	999.9	15.7	N/A	3.2	10.5
S of St. John (ocean)^b^	9/6/17 16:00	11.0	1006.9	21.9	N/A	5.6	6.9
Virgin Gorda, British Virgin Islands (landfall)^a^	9/6/17 16:30	37.0	915	79.7	N/A	N/A	N/A
Lameshur Bay, St. John^b^	9/6/17 17:36	2.5	945.1	N/A	N/A	N/A	N/A
S of St. John (ocean)^b^	9/6/17 19:00	11.0	1007.2	22.9	N/A	5.1	7.7
Trunk Bay, St. John^b^	9/6/17–9/7/17	6.5	N/A	N/A	171.45	N/A	N/A
S of St. John (ocean)^b^	Average from 9/6/17 7:00–9/7/17 23:00	11.0	1007.18	11.5	N/A	2.65	8.2
***Hurricane Jose***							
Peak Intensity E of St. John (ocean)^a^	9/9/17 0:00	645.0	938	69.45	N/A	N/A	N/A
NE of St. John (ocean)^a^	9/10/17 0:00	230.0	943	59.1	N/A	N/A	N/A
N of St. John (ocean)^a^	9/10/17 6:00	230.0	945	59.1	N/A	N/A	N/A
S of St. John (ocean)^b^	9/10/17 0:00	11.0	1008.4	4.4	N/A	1.4	13.3
S of St. John (ocean)^b^	9/10/17 6:00	11.0	1008	5.1	N/A	0.8	11.1
Trunk Bay, St. John^b^	9/9/17–9/11/17	6.5	N/A	N/A	0.00	N/A	N/A
***Hurricane Maria***							
Peak Intensity S of St. John (ocean)^a^	9/20/17 3:00	114.0	908	77.17	N/A	N/A	N/A
SE of St. John (ocean)^a^	9/20/17 6:00	91.0	913	72.02	N/A	N/A	N/A
S of St. John (ocean)^b^	9/20/17 3:00	11.0	998.5	19.6	N/A	5.1	10
S of St. John (ocean)^b^	9/20/17 5:00	11.0	995	18.3	N/A	5.5	11.8
S of St. John (ocean)^b^	9/20/17 6:00	11.0	993.1	20.3	N/A	6.7	11.1
S of St. John (ocean)^b^	9/20/17 7:00	12.0	992.5	19.7	N/A	7.9	11.1
Trunk Bay, St. John^b^	9/19/17–9/22/17	6.5	N/A	N/A	145.28	N/A	N/A
S of St. John (ocean)^b^	Average from 9/19/17 21:00–9/20/17 23:00	11.0	1001.93	14.8	N/A	4.6	9.8

Note that Hurricane Jose waves would not affect sediment transport, unlike Irma and Maria, because wave height is too low. Peak Intensity indicates the lowest pressures and highest winds of the storm’s lifetime. Barometric Pressure, Wind Speed, Wave Height, and Wave Period taken from NOAA buoy data and reports^52,54–56^. During Hurricane Irma the NOAA ocean buoy south of St. John was down immediately after the storm passed, no data was recorded during 17:00 and 18:00 on 9/6/17.

^a^Aerial measurement at storm center. ^b^Surficial measurement taken at sea level.

On September 10^th^, 2017, four days after Hurricane Irma hit St. John, Category 4 Hurricane Jose passed ~215 km northeast of the island^54^, though little rains, waves, or winds affected St. John (Table 1, Supplementary Fig. S1). Eight days later on September 20^th^, 2017, the eye of Category 5 Hurricane Maria passed ~90 km south of St. John, just 14 days after Hurricane Irma^55^ (Fig. 2). As Maria moved past St. John it reached its peak intensity of 77 m s^−1^ (Table 1)^55^. A buoy located ~11 km southwest of Coral Bay recorded maximum wave heights of 5.6 and 7.9 meters with maximum periods of 10.5 and 11.8 seconds for Irma and Maria, respectively^56^ (Table 1, Supplementary Fig. S1). Coral Bay opens to the Caribbean Sea in an east-south-east direction (Fig. 2). Thus, Coral Bay’s orientation is exposed to a direct impact from common Atlantic hurricane trajectories. Wave heights and periods during Jose were near average and likely did not affect the region due to low wave height^56^ (Table 1, Supplementary Fig. S1).

Understanding modern hurricane deposition is important for assessing impacts to coastal ecosystems and to accurately interpret the geologic record of past events. Furthermore, characterizing previous storm events is challenged by the difficulty in distinguishing a storm bed from a tsunami bed and vice versa^57,58^, despite excellent modern stratigraphic records^48,59^. In this study, we use a rare dataset to map and characterize the deposition that occurred following three major 2017 hurricanes (Irma, Jose, and Maria). We acquired multibeam bathymetry and sediment grab and core samples in Coral Bay shortly before and after the three storms. Modern multibeam bathymetry is particularly adept at resolving fine-scale differences in the nearshore environment, where techniques such as LiDAR are inadequate due to nearshore water turbidity^60^. Sediment samples provided crucial ground-truthing for pre- and post-storm multibeam surveys. This pre- vs. post-storm comparison is the first, to our best knowledge, to be collected in a tropical island setting. Our objectives are to use this rare dataset to map modern hurricane deposition and erosional patterns to better understand historical hurricane records, future depositional units and their potential impacts on the coastal system (Fig. 1).

## Results

### Pre/Post Storm Survey Difference Map

**Figure 3:**
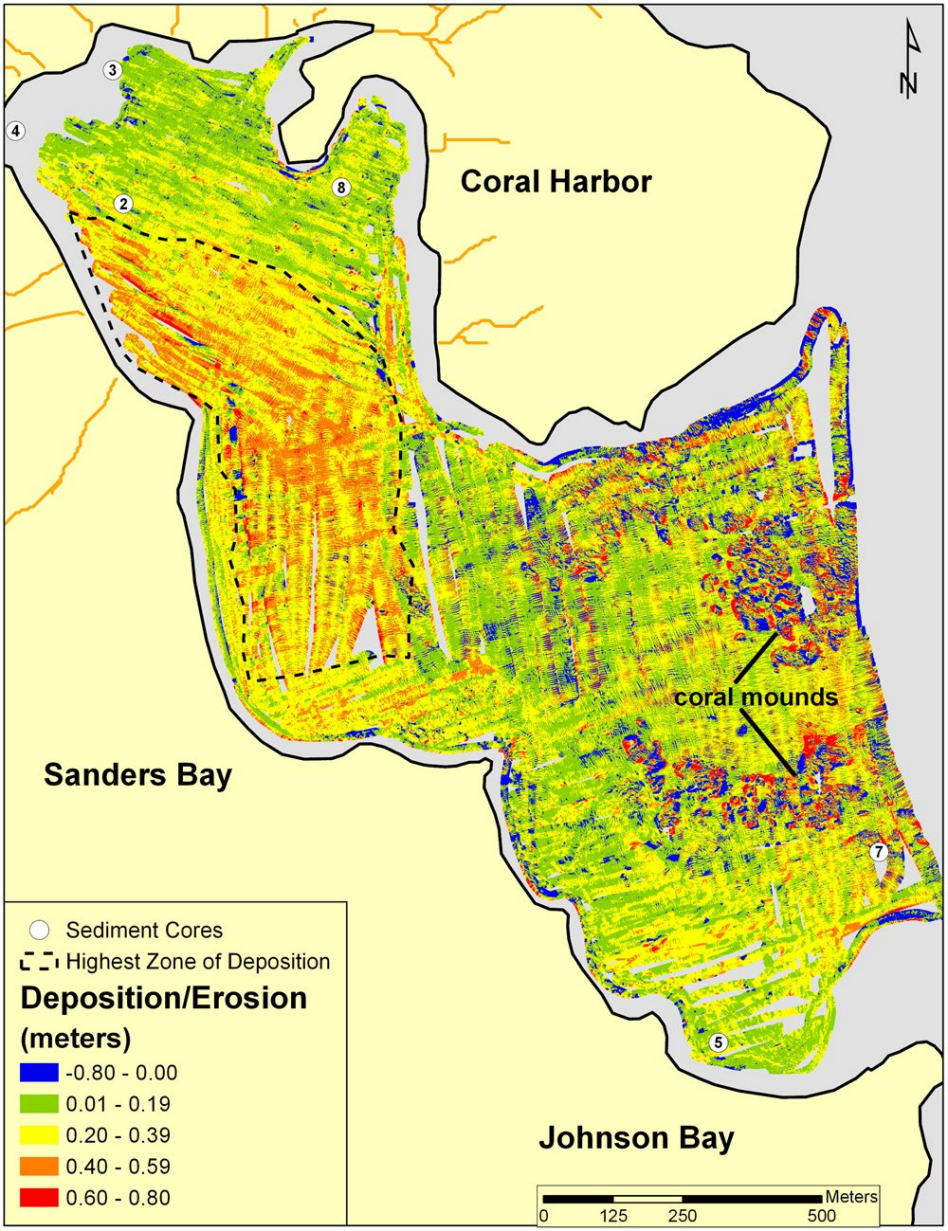
A difference map of Coral Bay, St. John, USVI. The post-storm November 2017 survey was subtracted from the pre-storm August 2017 survey. Deposition is indicated by positive values (green, yellow, orange, red); erosion in negative values (blue). The largest area of deposition (outlined with a black dashed line) is characterized by deposition ranging from 20 to 60 cm. Coral mounds in central Johnson Bay have a general flute mark pattern of deposition toward Coral Harbor and erosion to seaward. Six sediment cores (white circles) were collected in November of 2017.

The post-storm minus pre-storm multibeam bathymetry difference map (Fig. 3) shows widespread deposition throughout the 1.28 km^2^ coverage area. The depositional layer is fairly uniform (~+/−20 cm) in distribution, except an area of thick accumulation (+40–62 cm) in southwestern Coral Harbor and northwestern Sanders Bay (Fig. 3). Despite deposition throughout most of the survey area, some erosion occurred, especially around the perimeters of coral mounds (1–3 m in height) in the southern part of the survey area (Fig. 3). In particular, the mounds displayed a flute mark pattern of deposition toward Coral Harbor and erosion seaward (Fig. 3). The total calculated depositional volume is +322,317 m^3^ and the erosional volume is −29,329 m^3^ using the average determined vertical offset and a 10 cm grid spacing (Methods). If distributed evenly over the coverage area, the average thickness of the depositional unit would be ~25 cm. Considering uncertainties in the vertical offset, the average thickness of the depositional unit is 25 cm +/− 12 cm (Methods).

### Sediment Sample Analysis

**Figure 4:**
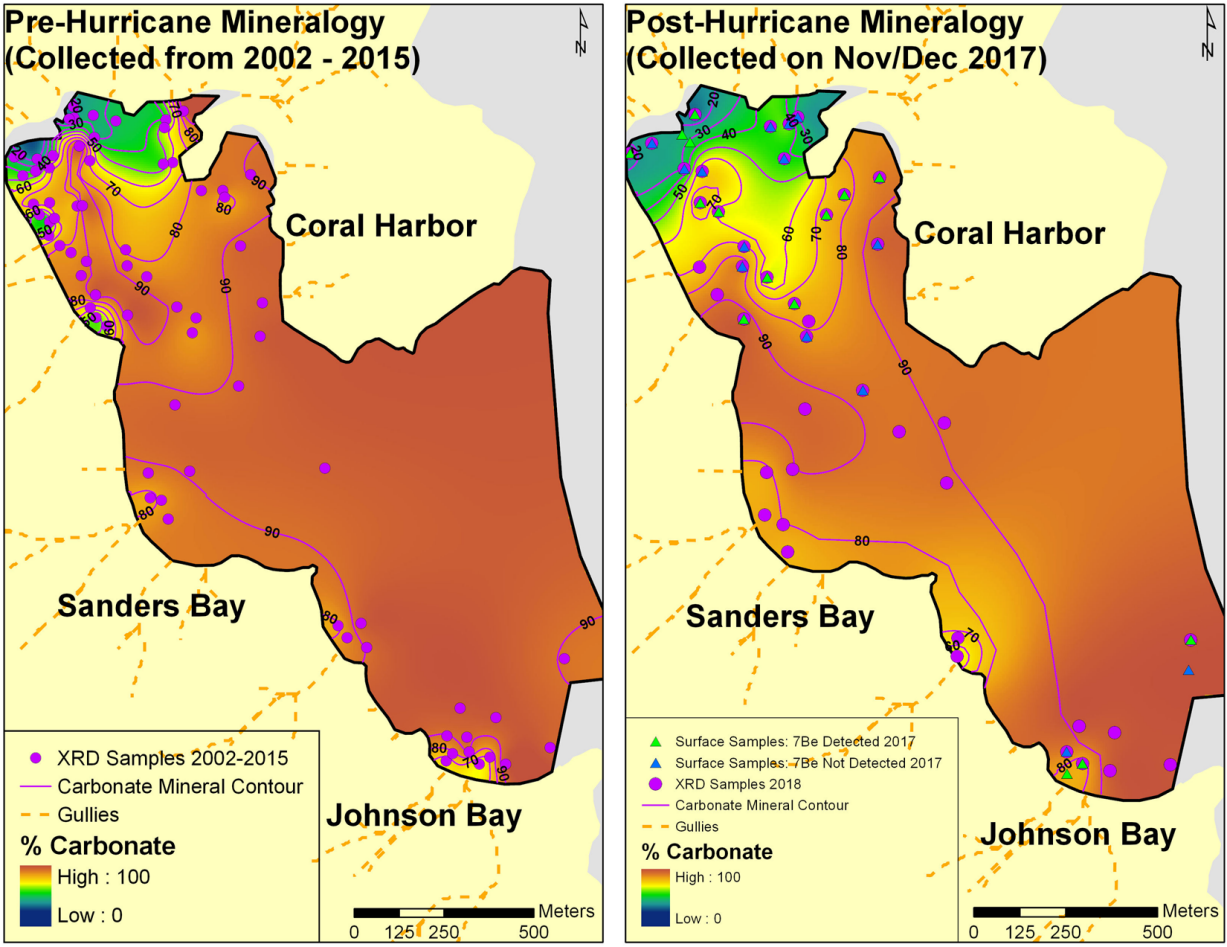
Surficial mineralogy of Coral Bay, St. John pre (left) and post (right) September 2017 Hurricanes Irma, Jose, and Maria. The post hurricane map also includes samples run for ^7^Be (triangles). Both pre- and post-storms, the seafloor was predominantly carbonate while there was some intrusion of terrestrial material following the hurricanes in northern Coral Harbor (right). Mineralogy was determined by XRD (X-Ray Diffraction).

**Figure 5:**
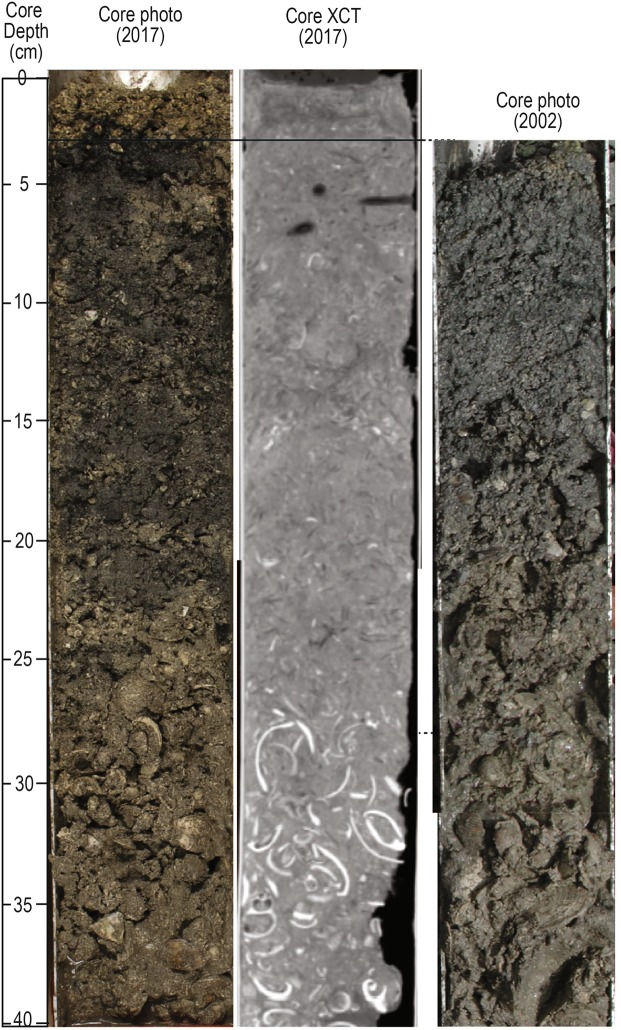
Core 2 collected in November 2017 (location in Fig. 4) showing a 3–4 cm thick surficial layer visible in the core photo (left) and XCT scan (middle). This layer was not present in a core taken in 2002 in the same location (right). This layer also contains Beryllium-7 indicating recent deposition (~months). X-Ray Computed Tomography (XCT).

^7^Be (half-life ~53 days) can be used to detect recent terrigenous sediment input (i.e., over the past ~1 year), and was found at 16 of 31 sample locations (cores and grabs) taken post storm (November 2017) (Fig. 4, Supplementary Table S1). As expected, presence of ^7^Be was observed near major gully effluences and not in the center of the bay (Fig. 4). One sample in south-central Johnson Bay had ^7^Be detected, but is proximal to a narrow seafloor channel (Figs 2 and 4). Of these, ^7^Be was detected in the six sediment cores collected in/near our survey area in November, 2017 (Fig. 3, Supplementary Table S1). Core 4 (Fig. 3) had ^7^Be from the core top downcore to ~1 cm. Two cores (2 and 5), detected ^7^Be down to 0.8 cm, and three cores (3, 7, and 8) detected ^7^Be down to at least 0.2 cm (Fig. 3, Supplementary Table S1). Also present in Core 2 was a capping unit extending ~4 cm downcore dominantly composed of coralline algae (*Halimeda* spp., indicating that it is primarily marine-derived) and mixed carbonate/terrestrial sands and silts (Figs 3 and 5). The location of Core 2 was previously core sampled in 2002^61^ and shows matching stratigraphy except for the capping 4 cm layer (Fig. 5).

The mineralogy of surface sediments both pre- and post-storms is dominated by marine-derived carbonate minerals (Fig. 4, Supplementary Table S2). High percentages (>80%) of marine carbonates persist away from the coast in Sanders and Johnson Bay both pre- and post-hurricanes Irma, Jose, and Maria. Isolated spots near the coast (gully effluences) have lower percentages of carbonate minerals (Fig. 4). Carbonate content generally decreased post-storm in the northern and middle parts of the Coral Harbor, but slightly increased in the southern region (Fig. 4).

## Discussion

Hurricanes Irma and Maria, and likely to a lesser extent Jose, resulted in a widespread depositional unit throughout western Coral Bay (Fig. 3). This is confirmed independently by pre- and post-storm multibeam bathymetry differencing and sediment sample analysis. The depositional layer varies in thickness, but in general is on the decimeter scale. Decimeter-scale storm beds are consistent with findings from other hurricane studies employing a range of methods^62–65^. Rapid-response studies that assess sedimentation and morphologic seafloor changes following major hurricanes are rare and have generally focused on the barrier island systems of the contiguous United States^64,66,67^. A common finding from pre- and post-storm analyses documents the potential for significant sediment transport and deposition in the marine environment, similar to other rapid and longer term studies focusing on storm deposition in coastal embayments^59,65,66^. In nearshore regions (1–15 m water depth), net deposition of 10–30 cm has been commonly observed with little erosion^7,62–66,68–71^. While erosion has been observed in some offshore settings (>15 m water depth)^21,62–64,66,72^. In a similar island setting to St. John, Kosciuch *et al*.^7^ found 10–44 cm thick deposits following Category 5 Tropical Cyclone Pam in embayments in Vanuatu in the southeast Pacific. Overall, our average of 25 cm depositional thickness with little erosion in a nearshore environment (0.5–17.5 m water depth) is consistent with other studies.

**Figure 6:**
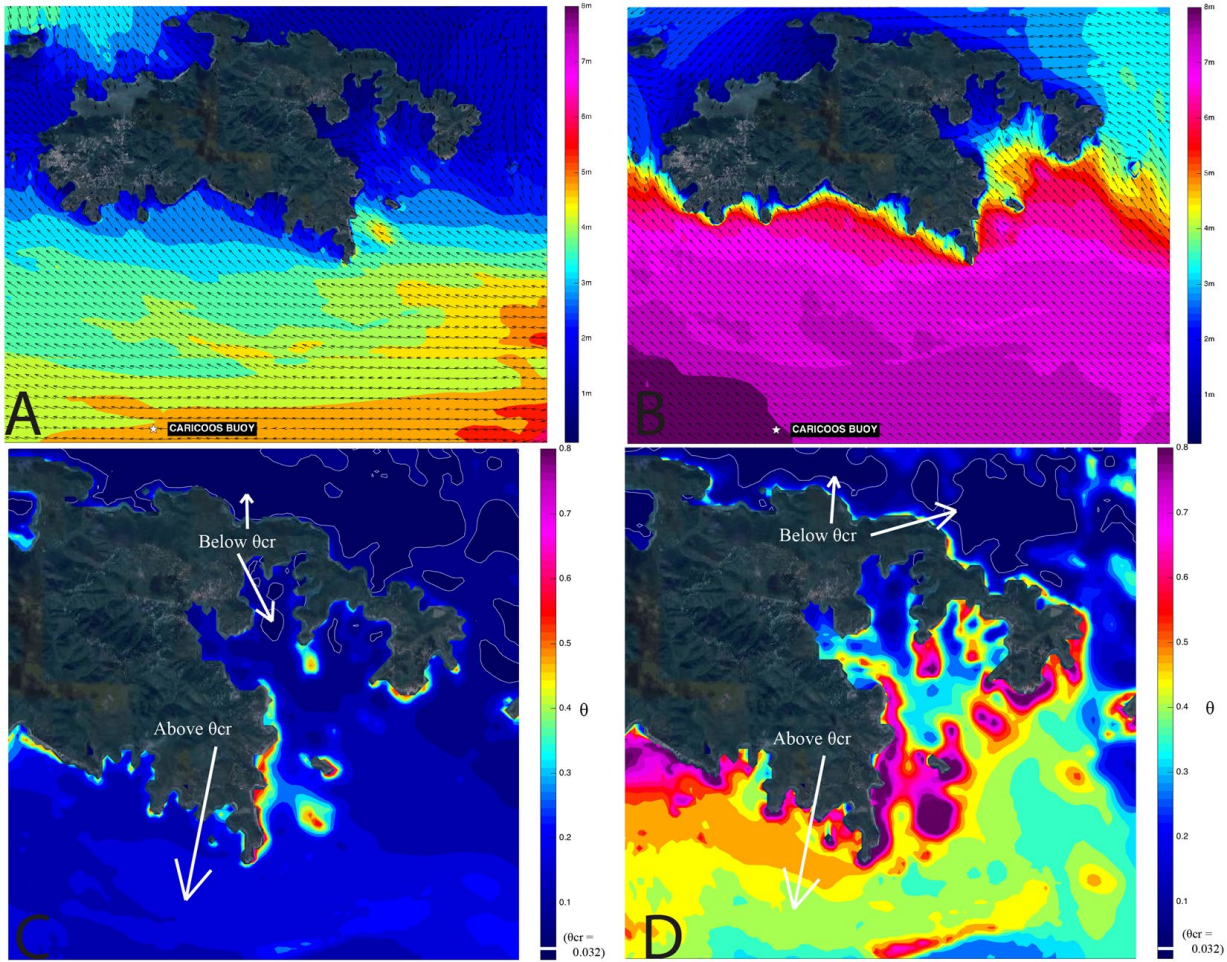
Calculated and modeled products from SWAN wave model in St. John, US Virgin Islands during Hurricane Irma (left) and Maria (right) in 2017. Panel A: Maximum wave height and direction during Hurricane Irma. Vector arrows show dominant wave direction. CARICOOS wave buoy location is noted in Table 1 (listed as “S. of St. John”) and in the text. Panel B: Maximum wave height and direction for Hurricane Maria. Vector arrows show dominant wave direction. Panel C: Maximum Shields parameter for Hurricane Irma. The white line represents the contour for critical Shields parameter (θ_crit_ = 0.032). Panel D: Maximum Shields parameter for Hurricane Maria. The white line represents the contour for critical Shields parameter (θ_crit_ = 0.032). (All image data shown are from Google, Digital Globe and generated using MatLabR2018b, https://www.mathworks.com/products/matlab.html).

We interpret that much of the net sediment volume increase in Coral Bay is due to marine sediment resuspension and transport caused by extreme wave action and strong wind and wave-induced currents. Sediment mobilization is dependent on many factors including: bay-bottom roughness, grain size and density, seawater density, wave height and period, water depth, and orbital velocity at the sea floor^73^. We modeled wave height and period using the SWAN wave model and estimated orbital velocities using linear wave theory (Methods). We assumed seabed roughness conservatively^74^ from previously measured^45^ medium-sized sand grains (0.05 cm diameter), and used the standard specific gravity for aragonite (2.93 g/cm3), which is a common carbonate mineral found in this region^43^ and in the deposit (Fig. 4, Table 2, and Supplementary Table S2). We combined these parameters with our modeled results to calculate the non-dimensional critical Shields parameter for both Hurricane Irma and Maria (Methods) (Fig. 6). The critical Shields parameter indicates the minimum non-dimensional shear stress needed for sediment mobilization^75^.

**Table 2:** Parameters used to calculate the critical Shields parameter during 2017 Hurricanes Irma and Maria for St. John, US Virgin Islands.

Assumed Variables	Assumed or Calculated Value	Units	Derived From
Bed Roughness	0.125	cm	Deigaard^73^
Grain Size (diameter)	0.05	cm	Brooks *et al*.^45^
Seawater Density	1025	kg/m^3^	Standard value
Sediment specific gravity	2.93	g/cm^3^	Standard value for aragonite
Seawater viscosity	1.05 × 10^−6^	m^2^/s	Standard value

Using the parameters mentioned above and standard values for seawater density and viscosity for the region (Table 2), the critical Shields parameter was estimated at 0.032 (Methods)^76^. Maximum non-dimensional shear stresses (Shields parameter) were then estimated over the southern St. John seafloor (Fig. 6) again using parameters derived in Table 2 and the wave model output (Methods). During both Irma and Maria the critical Shields parameter value was greatly exceeded in our coverage area and most areas south of St. John indicating the potential for sediment mobilization in the predominant wave direction (toward our coverage area) (Fig. 6). St. John sits on a wide, low-gradient shelf (30–60 m water depth) that extends ~12 km south of St. John, an area of ~42.3 km^2^ (Fig. 2). We find the critical Shields parameter was exceeded throughout all but northern Coral Bay during both Irma and Maria, with much larger values for Maria (Fig. 6). Thus, Irma was likely responsible for breaking up and resuspending shelf sediments finer than medium sand south of St. John, while Maria (with larger wave heights, periods, and higher Shields parameter values) was more likely to transport the sediments into the study area (Figs 2 and 6). Waves recorded during Jose were not significant in height and were likely too small to transport any significant amount of sediment (Supplementary Fig. S1). Mineralogical results are consistent with this interpretation due to the large amounts of the marine-derived, carbonate mineral aragonite found in our coverage area (Fig. 4, Supplementary Table S2). The aragonite likely comes from calcareous algae (possibly *Halimeda* spp., found in Coral Harbor^77^ and Core 2, Fig. 5) which dominantly exists from inner Coral Harbor to at least ~6 km offshore on the southern shelf of St. John^77,78^.

The recent nature of the large depositional event is confirmed with ^7^Be measurements of sediment grab and core samples. Half of the surficial sediment grab samples (~5 cm deep) contain ^7^Be, indicating recent deposition within the past year (Fig. 4). The downcore depth at which ^7^Be was detected, at least 0.8 cm in 3 cores, is representative of exceptionally high accumulation rates over the previous year^45,61^. The presence of ^7^Be can be diluted by large amounts of marine sediments, particularly coarse grained material as ^7^Be does not sorb well to large grains. The ^7^Be signal combined with surface mineralogy (Fig. 4) strongly suggest a recently deposited unit that is dominantly composed of marine-derived carbonate (Supplementary Table S2). However, ^7^Be only corroborates recent deposition and it may not be present throughout the new depositional layer. In fact, the layer change contact in Core 2 (~4 cm downcore), is 3.2 cm below detected ^7^Be (Fig. 5, Supplementary Table S1). The same location at Core 2 was previously cored in 2002^61^, but does not show the ~4 cm capping layer (Fig. 5). In this region, the pre- and post-hurricane bathymetric difference map shows deposition from 1–39 cm. However, it should be noted that handheld GPS units used for the core locations introduce positional inaccuracies. Thus, the area over which the core could have been taken can vary by several meters (possibly contributing to the large disparity in thickness).

Despite an annual amount of rainfall recorded between bathymetric surveys (~1,260 mm), watershed modeling (Methods) suggest that land-derived sediment contributed a trivial portion (0.1%) of the total volume of sediment deposited. Detection of ^7^Be downcore shows that terrestrial deposition occurred but when compared to the marine-derived component, is insignificant. Importantly, even when accounting for 100% sediment delivery to the coast (Methods, Supplementary Methods), estimated terrestrial inputs, including hurricane induced surface erosion, treethrow, and streambank erosion, equal less than 0.2% of the total estimated depositional volume estimated by the bathymetric surveys. We therefore interpret that nearly all of the total depositional volume is composed of marine-derived sediment, which could include some terrestrial sediment already in the marine environment. The transport and deposition occurred during a 14-day window between Hurricanes Irma, Jose, and Maria, but primarily during Irma and Maria. This is supported by our wave modeling (Fig. 6), surface sediment sample mineralogy (Fig. 4), and the marine carbonate fragments found in the upper 4 cm of Core 2 (Fig. 5).

The volume of hurricane-induced deposition that occurred during a 14-day span in 2017 is approximately equivalent to several centuries of sediment accumulation under typical conditions (including hurricanes). Core-derived sediment accumulation rates were previously estimated in Coral Bay by Brooks *et al*.^45,61^. They report an overall accumulation rate of 0.08 cm yr^−1^ during the last few thousand years and 0.20 cm yr^−1^ over the last 100 years. The increase is attributed primarily to anthropogenic development and the expansion of the unpaved road network in coastal watersheds since mid-20^th^ century^61^. The relative increase in sediment accumulation is similar to the sediment yields estimated for developed watersheds elsewhere in St. John^79,80^ (including our own sediment yield model). The average thickness of the new hurricane deposit (25 cm) equates to 313 years of pre-development sedimentation or 125 years of sediment accumulation under current conditions. The largest zone of deposition, on average ~50 cm thick (~263,000 m^2^ and ~130,000 m^3^; Fig. 3), equates to 625 years of sediment accumulation under pre-20^th^ century style land development regime and 250 years under current development and sedimentation patterns. This suggests that an equivalent of several hundreds of years of deposition (which include hurricane signals) occurred within a 2-week span.

Irma and Maria likely caused partial or full burial of seagrasses, corals, and calcareous algae in the study area. The substrate of Coral Harbor, Sanders Bay and the nearshore of Johnson Bay was primarily composed of seagrass and calcareous algae (*Thalassia* spp., *Syringodium* spp., *Halophila* spp., *Halodule* spp., and *Halimeda* spp.)^77,81^. Of the four seagrass genus dominantly found on St. John, all have shown partial mortality when experimentally buried to 5 cm and two species experienced full mortality when buried 10 cm for 60 days or more^31^. Given the average depositional thickness observed in Coral Bay(~25 cm) and that the survey occurred ~80 days after the last possible burial event (Maria) and ~104 days after the first possible burial event (Irma), it is possible that widespread mortality occurred. However, both the seagrass *Thalassia testudinum* and calcareous algae *Halimeda* spp. are resilient and communities have previously recovered from hurricane effects^25,31^.

We have characterized the short-term distribution and nature of the storm event but the long-term fate of the depositional unit is unclear. Natural processes may redistribute and/or erode the deposit. If the layer is preserved, compaction and burial will decrease the vertical thickness of the storm layer. Linear accumulation rates, such as those established by Brooks *et al*.^45,61^ do not account for gravity compaction, which this layer has not yet experienced and could ultimately lead to a smaller depositional thickness.

We assume that Irma and Maria primarily contributed to the new deposit, however, we find no obvious indicators distinguishing the individual contributions of each hurricane. We consider that the rapid succession of three major hurricanes, rather than if the storms were spread out over time, played a role in the ~322,317 m^3^ sediment deposit. Conceivably, Irma (the strongest storm) primed the system by disturbing and resuspending deeper marine sediments while Maria assisted in further eroding and transporting these sediments to the coastal reaches of Coral Bay. Regardless, this new volume originated under atypical conditions (3 major hurricanes in rapid succession) and thus normal coastal circulation may or may not adjust to redistribute this deposit.

Coral Bay is similar to many tropical volcanic systems both in the Caribbean and in the southwest Pacific. Due to the projected increase in the frequency of storms and their intensity^82,83^ understanding landscape response is important for future planning. Sediment derived from storms are a risk to critical coastal ecosystems such as coral reefs and should be considered in hurricane management plans and impact models.

## Methods

### Multibeam Surveying

We conducted two surveys in Coral Bay, St. John, USVI with a 7.6 m survey vessel using a R2Sonic^®^ 2020 multibeam echosounder equipped with varied power and internal motion and navigation (I2NS). An Applanix^®^ PosM/V navigational gyroscope paired with two L2 phase antennae recorded the position and motion data. The system was compression-mounted to the port side of the vessel with a pole mount and stabilized to prevent vibration and translation.

Data was collected using QPS^®^ QINsy hydrographic software and processed using Qimera. After the surfaces were cleaned, they were gridded to multiple cell sizes from 10 centimeters to 10 meters using the mean weighted average method.

A systematic error in depth occurred in the November survey. The shift was caused by a lack of additional GPS data, not allowing the coarse GPS and motion data to be post processed. To quantify the depth shift, the gridded surfaces were compared in a series of 2-D profiles across reef mounds in Johnson Bay whose elevation was not likely to change during the storms. In total 18 profiles and 5 structures were used to determine the offset. The average offset was −1.12 m with a maximum and minimum +/−0.12 m. It should be noted that the maximum offset yielded a depositional volume only 54% of the average offset depositional volume. The maximum offset still had an average depositional thickness of 10 cm. The minimum offset showed 30% greater deposition. The average offset was applied to the grid for the post-storm November dataset.

The November and August grids were differenced and each 0.10 m cell was assigned a single depth value. Each November 0.10 m grid cell was subtracted from the August survey 0.10 m grid cells creating a 0.10 m resolution difference map. Depositional volumes were calculated by summing the volumes of each individual cell in the difference map. As a check to the difference map, we examined several ~0.5 m deep depressions in Coral Harbor, which were observed in both surveys. Each depression profiled filled partially and had ~20–30 cm of deposition which correlates with the difference map, validating the average vertical offset of −1.12 m chosen for the November grid. We also assessed sensitivity to grid size spacing and depth uncertainty.

### Sediment Samples

During the post-storm field event, we acquired surficial sediment grab samples and sediment cores in both 3″ diameter aluminum barrels and 4″ polycarbonate tubes at the same location (Figs 3 and 4). The aluminum cores were scanned with X-Ray Computed Tomography (XCT) at a resolution of 0.625 mm per slice. The aluminum cores were then split longitudinally, photographed, and visually described. The polycarbonate cores were extruded vertically and subsampled at 0.5 cm intervals for short-lived radioisotopic analysis. Beryllium-7 (^7^Be) has a very short half-life (~53 days) and is an indicator of recent sediment deposition (~1 yr) and preservation of the core top. ^7^Be activities were measured on surface sediments and extruded core samples at 0.5 cm resolution on a GWL Series HPGe (High-Purity Germanium) Coaxial Well Photon Detector. Data were corrected for counting time, detector efficiency and geometry, as well as for the fraction of the total radioisotope measured yielding activity in dpm/g (disintegrations per minute per gram) (Supplementary Table S1). Detector efficiency was determined using similar methods to Kitto^84^ using the IAEA 447 standard. A calibration template was produced relating the counts measured to the known activity of the standard for the range of sample weights. By using the calibration template for various weights, self-absorption of the sample is included in the detector efficiency calculations. Surficial sediment samples were analyzed for mineralogic composition by powder X-Ray Diffraction (XRD) on a Bruker D4 Endeavor X-Ray diffractometer and processed using the Rietveld method^85,86^.

### Wave Modeling and Shields Parameter Calculation

A numerical simulation of the wave conditions associated with Hurricanes Irma and Maria was conducted in order to understand the potential for wave-induced sediment mobilization in and around Coral Bay. The simulation is based on the CARICOOS Nearshore Wave Model (CNWM), an operational model based on the Simulating WAves Nearshore (SWAN) spectral wave model which is described in detail in Booij *et al*.^87^. Additional details of the model setup and physics can be found in Anselmi *et al*.^88^, Canals *et al*.^89^, Canals and García^90^ and in Supplementary Methods. Wave conditions were simulated at very high spatial resolution (100 meters) in order to resolve wave transformation processes in the Virgin Islands shelf and in the complex coastline of St. John. A comparison between modeled and observed wave heights during the storms indicates the model captures the most important features of the wave events (Supplementary Fig. S1). Using the simulation results, we estimated maximum bottom shear stresses for each storm using the nondimensional Shields parameter formulation^75^ and compared the estimated values with the critical Shields parameter values that would be required for sediment mobilization (Supplementary Methods)^76^. We used standard values for seawater density and viscosity as well as locally observed grain sizes (Table 2)^45^. For grain density we used aragonite, a mineral dominantly found in Coral Bay^43^ (Supplementary Table S2). We used a Nikuradse roughness value proportional to the median sediment grain size (Supplementary Methods)^73^. It should be noted that, in reality, roughness values (and thus the associated shear stresses) would be much larger than the roughness corresponding to the median grain size, given the actual bedforms throughout the study site. However, in the absence of detailed information regarding physical bed roughness, using roughness values based on sediment grain size is a conservative estimate (and likely a significant underestimate) of the expected shear stresses.

### Watershed Sediment Budget Modeling

Estimates of terrestrial sediment contribution relied on the STJ-EROS model^80^. STJ-EROS is a GIS-based framework that relies on locally-derived functions^46,91^ and sediment delivery ratios (SDRs) to estimate net erosion and sediment yields into coastal waters. The model contains separate algorithms for natural and roaded surfaces. STJ-EROS is meant as an annual-based sediment budgeting tool, yet functions associated to surface erosion from undisturbed hillslopes and roads are driven by rainfall amounts, thus allowing model application to shorter periods. Additionally, two hurricane events occurred during the field testing of the model and delivered similar rainfall (Supplemental Methods). However, the model is unable to account for gullying (visually observed in 2017) or mass wasting (not observed in 2017). Treethrow contributions assumed an 8.5 Mg per linear km of stream per event. This is a conservative value considering that surveys that lead to the original estimate used by STJ-EROS (17 Mg per km per year) integrated treethrow caused by category 3 and 4 hurricanes (Hurricanes Hugo in 1989 and Georges in 1998)^92^ and not Category 5 Hurricanes like Irma and Maria. However, this estimate may be considered high as it presupposes that all of the sediment uprooted by this process is dislodged from the rootwad and thus available for further transport during the storm event that triggered its uprooting. Streambank erosion in STJ-EROS is also based on an annual rate, but since the amount of rainfall, and presumably runoff, associated to the period of interest equals the annual normal, we presumed that streambanks could have released an amount of sediment similar to the annual average. Required model input geodatabases represented a 2011 road survey^93^, a 3.9 km km^−2^ DEM-based stream network relying on a 0.1 ha source area threshold^50^ and a 2015 streambank field survey^94^.

The estimated sediment yield rate in STJ-EROS relies on user-defined SDRs, where SDR is the ratio of sediment delivered to the gross erosion occurring within the basin^95^. Given the small size and steep relief of catchments draining into Coral Bay (<10 km^2^), their SDR values are expected to be high. However, sediment delivery is confounded by the presence of coastal wetlands for which no empirical evidence exists on its sediment retention capacities. Therefore, watersheds draining through wetlands before delivering runoff into Coral Bay received a SDR value of 25%, while those directly draining into coastlines without an intervening wetland were assigned a SDR of 75%. The true sediment yield to coastal waters from all processes included in STJ-EROS likely lies between the model’s yield and net sediment production estimates.

The 7.3 km^2^ source area draining towards Coral Bay has a total of 27.8 km of gullies (i.e., ephemeral streams), but only of 4.9 km of those have erodible streambanks^94^. There are a total of 38.3 km of roads within the area draining into Coral Bay and 21.3 km of those are unpaved^94^. Only 29% of the source area is considered as having a high sediment delivery potential. Estimated sediment yield and total sediment produced for the period of interest are 280 and 560 Mg, respectively. The model estimates that these rates of sediment yield and production are 2.0 to 2.7 times above natural rates and that between 51% and 63% of this sediment is generated from unpaved roads.

## Supplementary information

Supplementary Material for Widespread Deposition in a Coastal Bay Following Three Major 2017 Hurricanes (Irma, Jose, and Maria)

## Data Availability

All data used for this study have been provided either in the text, supplementary material or are available through the National Science Foundation at www.data.gov (search our grant number, OCE-1762346).
