# Supplementary material for: Widespread Deposition in a Coastal Bay Following Three Major 2017 Hurricanes (Irma, Jose, and Maria)
Source: Sci Rep. 2019 May 8;9:7101. doi: 10.1038/s41598-019-43062-4 (PMC6506513; doi:10.1038/s41598-019-43062-4)
Supplement: Supplementary file 1 — Supplementary Material for Widespread Deposition in a Coastal Bay Following Three Major 2017 Hurricanes (Irma, Jose, and Maria) [file 41598_2019_43062_MOESM1_ESM.docx]

**Supplementary Material for:**

**Widespread Deposition in a Coastal Bay Following Three Major 2017 Hurricanes (Irma, Jose, and Maria)**

**Trevor N. Browning^1*^, Derek E. Sawyer^1^, Gregg R. Brooks^2^, Rebekka A. Larson^2^, and Carlos Ramos-Scharrón^3^ and Miguel Canals-Silander^4^**

^1^School of Earth Sciences, The Ohio State University, 125 S. Oval Mall, Columbus, Ohio 43210, USA, ^2^Department of Marine Science, Eckerd College, 4200 54^th^ Ave. S., St. Petersburg, Florida, 33711, USA, ^3^Department of Geography & the Environment, University of Texas at Austin, 305 E. 23^rd^ Street, Austin, Texas 78712, USA, ^4^UPRM Center for Applied Ocean Science and Engineering, Department of Engineering Sciences and Materials, University of Puerto Rico at Mayaguez, PR-108, Mayaguez, 00682, Puerto Rico

**Supplementary Methods**

*Wave Modeling Overview*

The wave modeling scheme used to simulate the temporal and spatial distribution of wave parameters for both Hurricane Irma and Hurricane Maria is based on the CARICOOS Nearshore Wave Model (CNWM), an operational model based on the Simulating Waves Nearshore (SWAN) spectral wave model which is described in detail in Booij et al.^1^ and in the SWAN user manual^2^. The simulations were run in third-generation mode and model physics include exponential wind wave growth and whitecapping, wave dissipation due to bottom friction, triad and quadruplet wave interactions, and wave refraction, diffraction and depth-induced breaking. Additional details of the model setup and physics can be found in Anselmi et al.^3^, Canals et al.^4^, and Canals & García^5^.

Grid resolution varied from 1 kilometer for the parent grid covering all of Puerto Rico and the US Virgin Islands (PRVI grid) to a child grid at 400-meter resolution covering the Virgin Islands shelf (VISH grid). Nested within this medium resolution grid is a third grid centered around the islands of St. Thomas and St. John (VIHR grid), with a spatial resolution of 100 meters. This last grid is able to resolve the wave processes around the complex coastline and bathymetry of St. John, including Coral Bay. The bathymetry used for the model is from the National Geophysical Data Center Digital Elevation Model (DEM) for Puerto Rico and the USVI^6^.

Boundary conditions for the model include spectral wave boundary conditions from the NOAA NCEP Multigrid WaveWatch III Model^7^ that are applied at the boundary of the PRVI grid. Surface wind forcing for both storms used output from the operational NOAA NCEP Hurricane Weather Research and Forecasting (HWRF) numerical weather model. The output of this model was generously provided to the authors by personnel from NOAA NCEP’s HWRF modeling division. The HWRF modeling system was developed by NOAA specifically for modeling hurricane wind and pressure fields (e.g. Zhang et al, 2016^8^).

Supplementary Figure S1 shows a comparison between the observed significant wave height at a CARICOOS buoy located south of St. John (Figure 6) and the simulated wave heights for both Hurricane Irma (September 4 – September 7^th^ 2017) and Hurricane Maria (September 18 – September 22 2017).

*Estimating maximum shear stresses*

The goal of the wave modeling presented here is to estimate whether there was potential for significant sediment suspension during Hurricanes Irma and Maria in the vicinity of Coral Bay. The potential for wave-induced sediment suspension during a storm is dominated by the turbulent shear stresses at the seabed^9^. If wave information is available at a given location, bed shear stresses can be estimated using near bed wave orbital velocities as follows^10^:

$\tau_{b}= \left( \frac{1}{2} \right)\rho f_{w}{U_{b}}^{2}$ *Equation* 1

where *τ_b_* is the bed shear stress associated with the wave conditions, *ρ* is the sea water density, *f_w_* is the wave friction factor, and *U_b_* is the near bed wave orbital velocity, given by^11^:

$U_{b}= \frac{\pi H}{T\sinh\left( kh \right)}$ *Equation* 2

where *H* is the local wave height (H=H_rms_=H_s_/$\sqrt{2}$), *T* is the peak period, *h* is the water depth and *k* = 2π/L, where *L* is the wave length obtained by solving the linear dispersion relation given by

$\omega^{2}=gk\tanh kh$ *Equation* 3

where $\omega=2\pi/T$ is the wave angular frequency and *g* is the gravitational constant. The wave friction factor *f_w_* is an important parameter for the computation of shear stresses and much research has been conducted on its measurement and estimation^9^. The wave friction factor will depend greatly on the seabed roughness, orbital velocities, and wave parameters such as the orbital excursion. The effects of the orbital excursion and the seabed roughness can be combined into the relative roughness parameter *r*, defined as

$$r=\frac{A_{w}}{k_{s}} , Equation 4$$

where $A_{w}$ is the length of the wave orbital excursion, defined as

$A_{w}=\frac{U_{b}T}{2\pi} ,$ *Equation* 5

A single equation for the wave friction factor was proposed by Roulund et al.^12^. This equation takes into account the expressions for *f_w_* by others^9,13,14^ for different ranges of the relative roughness parameter:

$f_{w}= \left\{ \begin{aligned} 0.32 \left( \frac{A_{w}}{k_{s}} \right)^{-0.8} 0.2< \left( \frac{A_{w}}{k_{s}} \right)<2.92 \\ 0.237 \left( \frac{A_{w}}{k_{s}} \right)^{-0.52} 2.92\leq\left( \frac{A_{w}}{k_{s}} \right)<727 \\ 0.04 \left( \frac{A_{w}}{k_{s}} \right)^{-0.25} \left( \frac{A_{w}}{k_{s}} \right)<2.92 \end{aligned} \right.$ *Equation* 6

For the present study we have used a Nikuradse roughness value proportional to the median sediment grain size $d_{50}$ following Fredsøe and Deigaard^14^:

$k_{s}=2.5d_{50}$ *Equation* 7

It should be noted that, in reality, roughness values (and thus the associated shear stresses) would be much larger than the roughness corresponding to the median grain size, given the actual bedforms throughout the study site. However, in the absence of detailed information regarding physical bed roughness, using roughness values based on sediment grain size is a conservative estimate (and likely a significant underestimate) of the expected shear stresses.

Once the bed shear stresses are estimated using the simulated wave parameters during the storm, it is necessary to determine if the maximum stresses were enough to generate significant sediment suspension. The Shields parameter^15^ ($\theta$) is a non-dimensional number that is widely used as an indicator for the initiation of motion of a given sediment particle:

$\theta= \frac{\tau_{b}}{(\rho_{s}-\rho)gd_{50}}$ *Equation* 8

where $\tau_{b}$is the bed shear stress and $\rho_{s}$ is the sediment density. For sediment suspension to occur, the Shields parameter must exceed a critical value, $\theta_{cr}$, that can be estimated as follows^9^:

$\theta_{cr}= \frac{0.30}{1+1.2 d_{*}}+ 0.055 \left[ 1-e^{-0.02d_{*}} \right]$ *Equation* 9

where *d_*_* is the non-dimensional sediment diameter, given by

$d_{*}=\left[ \left( s-1 \right)g/\upsilon^{2} \right]^{1/3}d_{50}$ *Equation* 10

where *s* is the sediment specific gravity and $\upsilon$is the water kinematic viscosity. For medium sized sand with $d_{50}$= 0.0005 m, s=2.93 g/cm^3^, the critical Shields parameter is approximately $\theta_{cr}$= 0.032. Finally, the actual values of the maximum Shields parameter we have estimated here were likely much larger given the conservative values we have used for the bed roughness.

*Watershed Sediment Budget Modeling Justification*

The STJ-EROS model is based on the sediment budget concept. A sediment budget represents an attempt to quantitatively describe sediment production, movement, and storage across landscapes^16^. Although its legitimacy has been critiqued due to the tendency of users to simplify controlling processes^17,18^, the sediment budget framework is deemed useful for geomorphic and watershed management purposes as it can isolate the individual contributions of different sediment sources within a watershed ^19-22^.

For the purposes of STJ-EROS, a landscape unit is defined as an area with a consistent set of erosion processes that produce sediment at spatially uniform rate^23^. Sediment production rates were measured from the following landscape units:

- erodible streambanks;
- stream margins subjected to soil disturbance by treethrow;
- undisturbed hillslopes and zero-order catchments;
- road travelways; and
- road cutslopes.

Measured rates were used to develop empirical prediction models and to assign those as spatially uniform sediment production rates for each landscape unit^24-26^.

To route sediment through a watershed it is necessary to quantify the rate of sediment movement among temporary storage sites^27^. To this day, modeling the transfer of sediment among different landscape units still remains largely unsatisfactory due to the complexities of accurately describing the processes involved in source-to-stream-to-watershed outlet connectivity^28,29^. In STJ-EROS, the efficiency by which terrestrial sediment is transferred to the marine environment is controlled by user-defined sediment delivery ratios (SDRs), where SDR is the ratio of sediment yield to the net erosion within the basin^30^. STJ-EROS refines this approach by allowing users to define specific areas within the modeled watersheds with different sediment delivery potentials. The classification of SDRs by delivery potential is a pragmatic compromise between an overly simplistic use of a singular watershed-scale SDR and a more physically based approach that requires much more detailed input data but that may not necessarily enhances accuracy^31,32^. The approach used in STJ-EROS is conceptually simple, easy to implement, computationally undemanding, and appropriate given the model objectives and its intended use by planners and resource managers.

*Watershed Sediment Budget Modeling Field Methods*

Algorithms representing sediment production rates from natural and anthropogenic sources in STJ-EROS were measured in St. John by a variety of field methods described in a Ph.D. dissertation^24^ and a series of articles^25,26,33^.

Streambank erosion at four stream reaches was quantified over a two-year period by erosion pins^34^. These four sites represent the approximately 17% of the assessed fluvial network where streams have incised through unconsolidated, poorly-sorted Quaternary alluvium^24,25^. In these incised reaches the streambanks are 0.6 to 2.3 m high, largely unvegetated, and show little layering. Loose, angular, gravel-sized fragments constitute about 25% of the deposits, and these coarse fragments are supported by a fine matrix consisting of approximately 25% sand and 50% silt and clay. Historical artifacts within some of these deposits indicate that the upper portions of the alluvium were deposited during the plantation and sugar-producing era in St. John (early 18^th^ to early 20^th^ century)^35^. The remaining 83% of the fluvial network consists of colluvial headwater channels that feed into first-order cascade or step-pool ephemeral channels^36^. These channels are ephemeral and are confined within steep ravines^37,38^. Minimal streambank erosion is expected from the colluvial headwater channels because they lack well-defined banks, or from the cascade and step-pool channels because these banks typically consist of boulders and largely unweathered bedrock.

In each study reach, five to nine 15-cm long erosion pins were installed along 2 to 4 vertical columns, yielding a total of 82 erosion pins. The length protruding from the bank was measured to the nearest millimeter at the time of installation and another 1-3 times at frequencies ranging from approximately six months to two years. The rate of streambank erosion or aggradation in centimeters per year was calculated from the net change in length over the time between measurements. These values were multiplied by the estimated bulk density to yield sediment production in Mg per hectare per year.

The number and volume of uprooted rootwads within approximately 3 m of the streams was assessed in early 2000 along 6.7 km of streams in three different watersheds (i.e., Fish, Lameshur, and Reef Bay). The volume of each rootwad was determined by measuring its diameter and thickness and assuming that its shape could be approximated by a cylinder. The percentage of the total rootwad volume that held uprooted soil was visually estimated for those rootwads that still held a significant amount of soil. The mean percent soil in the recent rootwads was assumed to be valid for the older rootwads. The condition of each rootwad was qualitatively described in terms of wood strength, bark condition, and the presence or absence of soil and small roots within the rootwad^39^.

The long-term rate of sediment delivery to the stream network by treethrow in Mg of soil per kilometer of stream per year was calculated with Eq. (1).

$Threethrow= \frac{\sum(Rootwad volume*Percent soil*Soil bulk density)}{(Channel length*Number of years)}$ *Equation* 11

The rootwad volume was in m^3^, and the soil bulk density was assumed to be 1.4 Mg m^−3^. The length of channel refers to the total length of the reach surveyed (in km), and the number of years is the period of time represented by the rootwads. Eq. (1) implicitly assumes that all of the soil in the rootwads along the streambanks is delivered to the stream network. We believe that this is a reasonable assumption because most of the streams in St. John are confined by steep hillslopes and the rootwads included in our field inventory generally were on top of vertical streambanks, indicating a very high probability of sediment delivery to the stream network. The number of years represented by our survey was the most difficult component to define, as this required estimating the age of the oldest rootwads and determining whether the frequency of rootwads observed during our field survey was representative of long-term conditions. The details of the rationale used to establish these values are in Ramos-Scharrón and MacDonald^25^.

Sediment production rates were periodically measured from 21 unpaved road segments between July 1998 and April 2000, and two of these segments continued to be monitored through November 2001^26^. To the extent possible, the segments were selected to represent a wide range of surface areas and slopes, as road segment area times slope is a useful surrogate for the tractive forces due to infiltration-excess overland flow on the road surface^40^. The mean width of the road segments was 4.7 m and the mean road surface area–including both the active travelway and the inside ditch–was 850 m^2^. The mean slope of the road segments was 10% and the range was from 1% to 21%. Road use was stratified into three classes: (a) roads exclusively used by light vehicles; (b) roads with light vehicle traffic plus four to six medium-sized delivery truck passes per day; and (c) abandoned roads. The a priori classification of road segments into these classes provided a secondary criterion for site selection. Time since construction or grading was not a primary criterion because all of the recently-constructed road segments were privately owned, the grading history was not always known when the road segments were being selected, and we had no control on when regrading occurred.

Road tread sediment production was measured by sediment fences placed immediately below a point of concentrated road drainage such as a cemented swale, unprotected cross-dip or culvert. Fences were constructed by attaching filter fabric to pieces of rebar hammered vertically into the ground^41^. The sediment trapped in the fences was collected and weighed in the field to the nearest ¼ kg. Samples of the trapped sediment were collected and used to determine percent moisture^42^. These data were used to convert the field-measured wet weights to a dry mass.

Eighty sediment production measurements were obtained during the study period, and the precipitation associated with each of the 80 measurements was obtained from one of the four recording rain gauges (Fig. 1). The study period included rainfall associated to Hurricane Georges in 1998 (50 - 100 mm total) and Hurricane Lenny (~140 mm total)^26^. Sediment production rates for individual road segments can be calculated as the product of total sediment production–normalized by road contributing area and rainfall–times any rainfall total. Sediment production was related to precipitation, surface area, slope, traffic, and grading history by graphical analysis and multiple linear regression.

Fences collecting sediment from the road tread (described above) captured material from both the road tread and the cutslope. To separate these two components, eight sediment fences were installed at the base of cutslopes in the Maho Bay area and along John Head Road in Catherineberg Estate. The eight cutslopes were nearly vertical, 1.2 to 4.2 m high, and had less than 10% vegetation cover. The mean surface area was 16 m^2^. Two of the cutslopes were exclusively composed of residual soil, two were dominated by slightly-weathered bedrock, and the remaining four cutslopes were dominated by moderately-weathered bedrock. Twenty measurements were taken between July 1998 and November 2001 at intervals ranging from a few months to just over a year. A visual classification system and detailed sketch maps were used to estimate the proportion of cutslope sediment that was delivered to the outlet of 20 road segments with sediment fences. A delivery potential of 75% was assumed for cutslope sections that had ditches or concentrated flowpaths at their toe, as the presence of depositional aprons indicated that sediment delivery rates were less than 100%. Cutslopes that were less than 3 m from the road tread but not delivering sediment directly to a ditch or concentrated flowpath were assumed to have a delivery potential of 10%. Zero delivery was assumed for cutslopes located more than 3 m from a ditch or flowpath. For each road segment, the amount of sediment from cutslopes was assumed to equal the product of the mean sediment production rate from cutslopes times the cutslope contributing area times the assumed delivery ratio^25^.

Sediment production rates at the catchment-scale were measured from July 1998 to November 2001 also with sediment fences. Sediment production rates from undisturbed areas were measured for four and two zero- and first-order catchments, respectively. Sediment fences were also installed on two first-order catchments that were receiving sediment from unpaved roads. The drainage areas of these catchments ranged from 0.9 to 15 ha, and the average hillslope gradients ranged from 15% to 37%. Each of these catchments had a dense cover of moist evergreen forest.

*Sediment Production Algorithm*

The measured sediment production rates for the different landscape units ranged over five orders of magnitude (Supplementary Figure S2; Supplementary Table S3). The mean streambank erosion rate was 100 Mg ha^-1^ yr^-1^. Uprooting of trees along stream margins was estimated to deliver 0.17 Mg of sediment per kilometer of stream per year, or 0.11 Mg ha^-1^ yr^-1^ for a 15-m wide stream corridor. This rate is just slightly lower than the value of 0.20 Mg ha^-1^ yr^-1^ reported from the Luquillo Experimental Forest (LEF) in eastern Puerto Rico^43^.

Surface erosion rates from unpaved road segments depend on rainfall, road slope to the 1.5 power, and frequency of grading (Supplementary Figure S2; Supplementary Table S3)^26^. Sediment production rates for road segments that were graded at least once every two years ranged from 6 Mg ha^-1^ yr^-1^ for a segment with a mean slope of 1% to 580 Mg ha^-1^ yr^-1^ for a segment with a mean slope of 21%. Ungraded roads had sediment production rates ranging from 51 to 140 Mg ha^-1^ yr^-1^ for segments with a mean slope of 10% and 16%, respectively. Abandoned road segments with a slope of 15% had a mean erosion rate of only 11 Mg ha^-1^ yr^-1^.

Cutslope sediment production rates ranged from 20 to 170 Mg per ha of cutslope surface per year, but accounted for only 9% of the sediment yield from unpaved roads at the road-segment scale^24^. The low sediment yield from cutslopes is due to their relatively coarse texture and the resulting low transport rates, and because the road runoff tended to be concentrated in tire tracks rather than in an inside ditch at the toe of the cutslopes^26,33^.

The mean sediment yield for undisturbed hillslopes was 0.01 Mg ha^-1^ yr^-1^, or about an order of magnitude less than the 0.10 – 0.50 Mg ha^-1^ yr^-1^ reported for undisturbed hillslopes in the LEF in Puerto Rico^44^. The lower value for St. John can be attributed to 60% less rainfall and a higher abundance of coarse rock fragments^25^.

*Watershed Sediment Budget Model Overview*

STJ-EROS calculates the amount of sediment from different sources that reaches the marine environment on a watershed scale, and uses GIS software to do so on a spatially explicit basis. The STJ-EROS model has six input routines and five routines that calculate sediment production and delivery (Supplementary Figure S3). The six input routines have user interfaces that allow the user to adjust some of the key variables controlling sediment production and delivery. The remaining five routines use pre-set erosion rates, user defined variables, and item values stored in nine data layers to calculate watershed scale sediment yields. A more complete description of the GIS data layers needed to run the model, flowcharts of the most important routines, and the program code can be found in Ramos-Scharrón (2004)^24^.

The STJ-EROS model was applied to three basins on St. John: Lameshur Bay, Fish Bay, and Cinnamon Bay, as part of a previous study^23^. In the absence of specific data on the sediment trapping efficiency of coastal wetlands, areas with a high potential for sediment delivery were assigned a SDR of 75%, while areas with an intervening coastal wetland filtering watershed inputs were categorized as having a moderate potential with a SDR of 25%. Predicted sediment yields under natural (undisturbed) conditions ranged from 2 to 7 Mg km^-2^ yr^-1^, while yield rates for contemporary (disturbed) conditions ranged from 8 to 46 Mg km^-2^ yr^-1^. Unpaved roads are estimated to increase sediment delivery rates by 3 – 9 times above background levels. Predicted basin-scale sediment yields for both undisturbed and current conditions are within the range of measured sediment yields and bay sedimentation rates in St. John (e.g., Brooks et al., 2007^45^). Unpaved roads were undoubtedly the dominant source of sediment accounting for up to 85% of the total estimated sediment yield, particularly for watersheds with high unpaved road densities. Streambank erosion proved to be the dominant natural sediment source.

| **Sample ID** | **Top Depth of interval (cm)** | **Bottom Depth of interval (cm)** | **Sample Type** | **^7^Be Activity (dpm/g)** | **^7^Be Activity error (dpm/g)** | **^7^Be Activity (dpm/g), Decay Corrected** | **^7^Be Activity error (dpm/g), Decay Corrected** |
| --- | --- | --- | --- | --- | --- | --- | --- |
| CB-17-SS-01 | N/A | N/A | Surface Grab Sample | 0.49 | 0.11 | 3.30 | 0.72 |
| CB-17-SS-03 | N/A | N/A | Surface Grab Sample | 0.00 | 0.00 | 0.00 | 0.00 |
| CB-17-SS-04 | N/A | N/A | Surface Grab Sample | 0.00 | 0.00 | 0.00 | 0.00 |
| CB-17-SS-05 | N/A | N/A | Surface Grab Sample | 0.18 | 0.02 | 0.48 | 0.05 |
| CB-17-SS-06 | N/A | N/A | Surface Grab Sample | 0.00 | 0.00 | 0.00 | 0.00 |
| CB-17-SS-08 | N/A | N/A | Surface Grab Sample | 0.16 | 0.02 | 0.65 | 0.09 |
| CB-17-SS-09 | N/A | N/A | Surface Grab Sample | 0.12 | 0.02 | 0.72 | 0.09 |
| CB-17-SS-10 | N/A | N/A | Surface Grab Sample | 0.00 | 0.00 | 0.00 | 0.00 |
| CB-17-SS-11 | N/A | N/A | Surface Grab Sample | 0.00 | 0.00 | 0.00 | 0.00 |
| CB-17-SS-12 | N/A | N/A | Surface Grab Sample | 0.00 | 0.00 | 0.00 | 0.00 |
| CB-17-SS-13 | N/A | N/A | Surface Grab Sample | 0.00 | 0.00 | 0.00 | 0.00 |
| CB-17-SS-14 | N/A | N/A | Surface Grab Sample | 0.00 | 0.00 | 0.00 | 0.00 |
| CB-17-SS-15 | N/A | N/A | Surface Grab Sample | 0.09 | 0.02 | 0.42 | 0.07 |
| CB-17-SS-16 | N/A | N/A | Surface Grab Sample | 0.06 | 0.01 | 0.18 | 0.04 |
| CB-17-SS-17 | N/A | N/A | Surface Grab Sample | 0.00 | 0.00 | 0.00 | 0.00 |
| CB-17-SS-18 | N/A | N/A | Surface Grab Sample | 0.00 | 0.00 | 0.00 | 0.00 |
| CB-17-SS-20 | N/A | N/A | Surface Grab Sample | 0.00 | 0.00 | 0.00 | 0.00 |
| CB-17-SS-21 | N/A | N/A | Surface Grab Sample | 0.00 | 0.00 | 0.00 | 0.00 |
| CB-17-SS-22 | N/A | N/A | Surface Grab Sample | 0.18 | 0.03 | 1.05 | 0.15 |
| CB-17-SS-23 | N/A | N/A | Surface Grab Sample | 0.27 | 0.03 | 1.98 | 0.21 |
| CB-17-SS-24 | N/A | N/A | Surface Grab Sample | 0.05 | 0.01 | 0.22 | 0.05 |
| CB-17-SS-26 | N/A | N/A | Surface Grab Sample | 0.00 | 0.00 | 0.00 | 0.00 |
| CB-17-SS-62 | N/A | N/A | Surface Grab Sample | 0.00 | 0.00 | 0.00 | 0.00 |
| CB-17-SS-86 | N/A | N/A | Surface Grab Sample | 0.14 | 0.02 | 0.88 | 0.11 |
| CB-17-SS-87 | N/A | N/A | Surface Grab Sample | 0.00 | 0.00 | 0.00 | 0.00 |
| Core 2 | 0.0 | 0.2 | Core Sample | 1.80 | 0.15 | 2.28 | 0.19 |
| Core 2 | 0.2 | 0.4 | Core Sample | 0.17 | 0.01 | 0.26 | 0.02 |
| Core 2 | 0.4 | 0.6 | Core Sample | 0.07 | 0.01 | 0.17 | 0.02 |
| Core 2 | 0.6 | 0.8 | Core Sample | 0.05 | 0.01 | 0.15 | 0.02 |
| Core 3 | 0.0 | 0.2 | Core Sample | 0.64 | 0.08 | 0.85 | 0.11 |
| Core 3 | 0.2 | 0.4 | Core Sample | 0.06 | 0.01 | 0.10 | 0.01 |
| Core 4 | 0.0 | 0.2 | Core Sample | 1.47 | 0.16 | 1.96 | 0.21 |
| Core 4 | 0.2 | 0.4 | Core Sample | 0.10 | 0.01 | 0.16 | 0.02 |
| Core 4 | 0.4 | 0.6 | Core Sample | 0.25 | 0.02 | 0.42 | 0.03 |
| Core 4 | 0.6 | 0.8 | Core Sample | 0.09 | 0.01 | 0.27 | 0.03 |
| Core 4 | 0.8 | 1.0 | Core Sample | 0.10 | 0.01 | 0.34 | 0.04 |
| Core 5 | 0.0 | 0.2 | Core Sample | 1.06 | 0.10 | 1.34 | 0.13 |
| Core 5 | 0.2 | 0.4 | Core Sample | 0.17 | 0.01 | 0.25 | 0.02 |
| Core 5 | 0.4 | 0.6 | Core Sample | 0.04 | 0.01 | 0.11 | 0.02 |
| Core 5 | 0.6 | 0.8 | Core Sample | 0.05 | 0.01 | 0.16 | 0.02 |
| Core 7 | 0.0 | 0.2 | Core Sample | 0.86 | 0.08 | 1.21 | 0.11 |
| Core 8 | 0.0 | 0.2 | Core Sample | 0.44 | 0.06 | 0.59 | 0.08 |

Supplementary Table S1. Detections of ^7^Be Data in surface sediment samples in Coral Bay, St. John, USVI after the 2017 Atlantic Hurricane season (November 2017). Surface samples represent sediment depths from 0 – 10 cm below the surface. Disintegrations per minute per gram (dpm/g).

| **Sample ID** | **Albite** | **Aragonite** | **Calcite** | **High Mg-Calcite** | **Quartz** | **Weighted R** | **Terrestrial** | **Carbonate** |
| --- | --- | --- | --- | --- | --- | --- | --- | --- |
| CB-17-SS-01 | 6.8 | 66.7 | 3.2 | 7.9 | 15.4 | 8.06 | 22.2 | 77.8 |
| CB-17-SS-02 | 0 | 81.9 | 5.1 | 12 | 1 | 7.45 | 1 | 99 |
| CB-17-SS-04 | 25.3 | 28 | 1.7 | 2.4 | 42.5 | 6.97 | 67.8 | 32.1 |
| CB-17-SS-05 | 14.7 | 54.6 | 1.8 | 2.7 | 26.2 | 6.77 | 40.9 | 59.1 |
| CB-17-SS-06 | 11.1 | 59.6 | 2.6 | 3.4 | 23.3 | 7.46 | 34.4 | 65.6 |
| CB-17-SS-10 | 26.8 | 22.5 | 0 | 1.2 | 49.5 | 8.42 | 76.3 | 23.7 |
| CB-17-SS-11 | 21.1 | 34.8 | 2.4 | 3.8 | 37.9 | 6.53 | 59 | 41 |
| CB-17-SS-12 | 19.7 | 37.8 | 2.4 | 2.2 | 37.9 | 7.58 | 57.6 | 42.4 |
| CB-17-SS-13 | 16.3 | 40 | 4 | 3.7 | 36 | 6.61 | 52.3 | 47.7 |
| CB-17-SS-14 | 30.2 | 20.7 | 2.5 | 4.8 | 41.8 | 6.21 | 72 | 28 |
| CB-17-SS-15 | 8.5 | 58.1 | 4 | 12.9 | 16.5 | 6.71 | 25 | 75 |
| CB-17-SS-16 | 4.5 | 70.1 | 3.8 | 12.9 | 8.6 | 7.1 | 13.1 | 86.8 |
| CB-17-SS-17 | 2.7 | 82 | 2.5 | 8.1 | 4.6 | 6.87 | 7.3 | 92.6 |
| CB-17-SS-18 | 5.8 | 68.2 | 2.8 | 10.6 | 12.7 | 7.44 | 18.5 | 81.6 |
| CB-17-SS-19 | 7.2 | 61.5 | 4.1 | 12.4 | 14.8 | 7.17 | 22 | 78 |
| CB-17-SS-20 | 9.8 | 55.7 | 4 | 10.7 | 19.9 | 7.24 | 29.7 | 70.4 |
| CB-17-SS-21 | 3.9 | 72.6 | 4.3 | 9.1 | 10 | 7.01 | 13.9 | 86 |
| CB-17-SS-22 | 2 | 89.7 | 1.1 | 3.6 | 3.7 | 8.53 | 5.7 | 94.4 |
| CB-17-SS-23 | 17.4 | 37.2 | 4.3 | 16.8 | 24.3 | 6.48 | 41.7 | 58.3 |
| CB-17-SS-24 | 7.6 | 61.8 | 3.6 | 11.2 | 15.8 | 7.01 | 23.4 | 76.6 |
| CB-17-SS-26 | 3.3 | 74.8 | 3.5 | 10.4 | 8 | 7.35 | 11.3 | 88.7 |
| CB-17-SS-27 | 2.5 | 83.4 | 1.7 | 6.8 | 5.6 | 7.42 | 8.1 | 91.9 |
| CB-17-SS-28 | 3.9 | 77.6 | 3 | 8.8 | 6.7 | 6.63 | 10.6 | 89.4 |
| CB-17-SS-29 | 8.2 | 65.1 | 1.8 | 11.1 | 13.8 | 7.09 | 22 | 78 |
| CB-17-SS-30 | 8.7 | 68.6 | 2 | 9.3 | 11.4 | 6.98 | 20.1 | 79.9 |
| CB-17-SS-31 | 8.7 | 58.7 | 2.2 | 14.1 | 16.2 | 7.15 | 24.9 | 75 |
| CB-17-SS-32 | 17.5 | 49.2 | 1.6 | 6.9 | 24.8 | 6.71 | 42.3 | 57.7 |
| CB-17-SS-33 | 12.6 | 57.8 | 1.8 | 8.4 | 19.4 | 6.71 | 32 | 68 |
| CB-17-SS-36 | 3.5 | 69.3 | 3.6 | 16.7 | 6.9 | 7.43 | 10.4 | 89.6 |
| CB-17-SS-37 | 3.1 | 75.6 | 2.9 | 10.4 | 8 | 7.9 | 11.1 | 88.9 |
| CB-17-SS-38 | 4.4 | 72.9 | 3.3 | 13.8 | 5.6 | 7.89 | 10 | 90 |
| CB-17-SS-78 | 10.1 | 62.8 | 3.1 | 4.8 | 19.2 | 7.08 | 29.3 | 70.7 |
| CB-17-SS-79 | 28.8 | 18 | 0 | 0 | 53.2 | 7.73 | 82 | 18 |
| CB-17-SS-80 | 29.8 | 11.9 | 0 | 0.7 | 57.6 | 7.28 | 87.4 | 12.6 |
| CB-17-SS-81 | 7.5 | 59.4 | 4.6 | 13.1 | 15.4 | 7.56 | 22.9 | 77.1 |
| CB-17-SS-83 | 10.3 | 64.7 | 2.1 | 3.9 | 19 | 6.93 | 29.3 | 70.7 |
| CB-17-SS-84 | 3.5 | 78.8 | 2.4 | 6.1 | 9.2 | 7.62 | 12.7 | 87.3 |
| CB-17-SS-85 | 7.9 | 67.4 | 2 | 9.1 | 13.5 | 6.54 | 21.4 | 78.5 |
| CB-17-SS-87 | 3.4 | 73.4 | 2.7 | 11.6 | 8.9 | 7.28 | 12.3 | 87.7 |
| CB-17-SS-88 | 0 | 79.3 | 2.2 | 16.1 | 2.4 | 7.6 | 2.4 | 97.6 |
| CB-17-SS-89 | 0 | 73.8 | 3.1 | 19.4 | 3.7 | 8.11 | 3.7 | 96.3 |
| CB-17-SS-90 | 0 | 78.3 | 3.5 | 14.1 | 4.2 | 7.32 | 4.2 | 95.9 |
| CB-17-SS-91 | 0 | 79.4 | 2.3 | 16.2 | 2.1 | 8.01 | 2.1 | 97.9 |

Supplementary Table S2. Mineralogy percentages (X-Ray Diffraction, Rietveld analysis) of surface sediment samples collected in November of 2017 in Coral Bay, St. John, USVI. Surface samples represent 0 – 10 cm of sediment below the seafloor.

| Sediment source | **Sediment production**  **(kg m^-2^ yr^-1^)** | Sediment production function(kg) |
| --- | --- | --- |
|  |  |  |
| Streambanks | 10 | [10] * 2 * *channel length with erodible banks** *bank height* * years |
| Treethrow | 0.01  [0.17 kg m^-1^ yr^-1^] | [0.17] * *channel length* * years |
| Undisturbed hillslopes | 0.001 | [ 6.4 x 10^-5^] * 14% rainfall * *area* *  [**1 + 3.4 * 0.004^†^**] |
| Graded roads | - 1. – 52   (slopes from 1% to 21%) | [-0.432 + 4.73 * *slope*^1.5^ * rainfall] * *road length* * *width* *  [**1 + 3.4 * 0.06^†^**] |
| Ungraded roads | 1. – 20   (slopes from 1% to 21%) | [-0.432 + 1.88 * *slope*^1.5^ * rainfall] * *road length* * *width* *  [**1 + 3.4 * 0.04^†^**] |
| Abandoned roads | 0.08 – 1.7  (slopes from 1% to 21%) | [0.071] * *slope* * rainfall * *road length* * *width* *  **1 + 3.4 * 0.001^†^** |
| Cutslopes | 0.0 – 5.7 | [0.09] * road segment sediment production |
|  |  |  |

Supplementary Table S3. Sediment production rates and empirical prediction functions for the different landscape units in STJ-EROS. Lengths, widths, and heights are in meters, time is in years, slope is a decimal, area is in m^2^, and rainfall is in centimeters. Empirical sediment production functions are in square brackets; corrections for the loss of silt-sized particles are bolded. Items in italics are taken from GIS data layers, underlined items indicate user-defined variables, and rainfall is calculated by multiplying the user-defined annual rainfall rate times the time in years. Road surface erosion accounts for 91% of the sediment yield from road segments, and cutslopes account for the remaining 9%. ^†^Refers to the percent of silt from Table 2 in Ramos-Scharrón and MacDonald^23^.


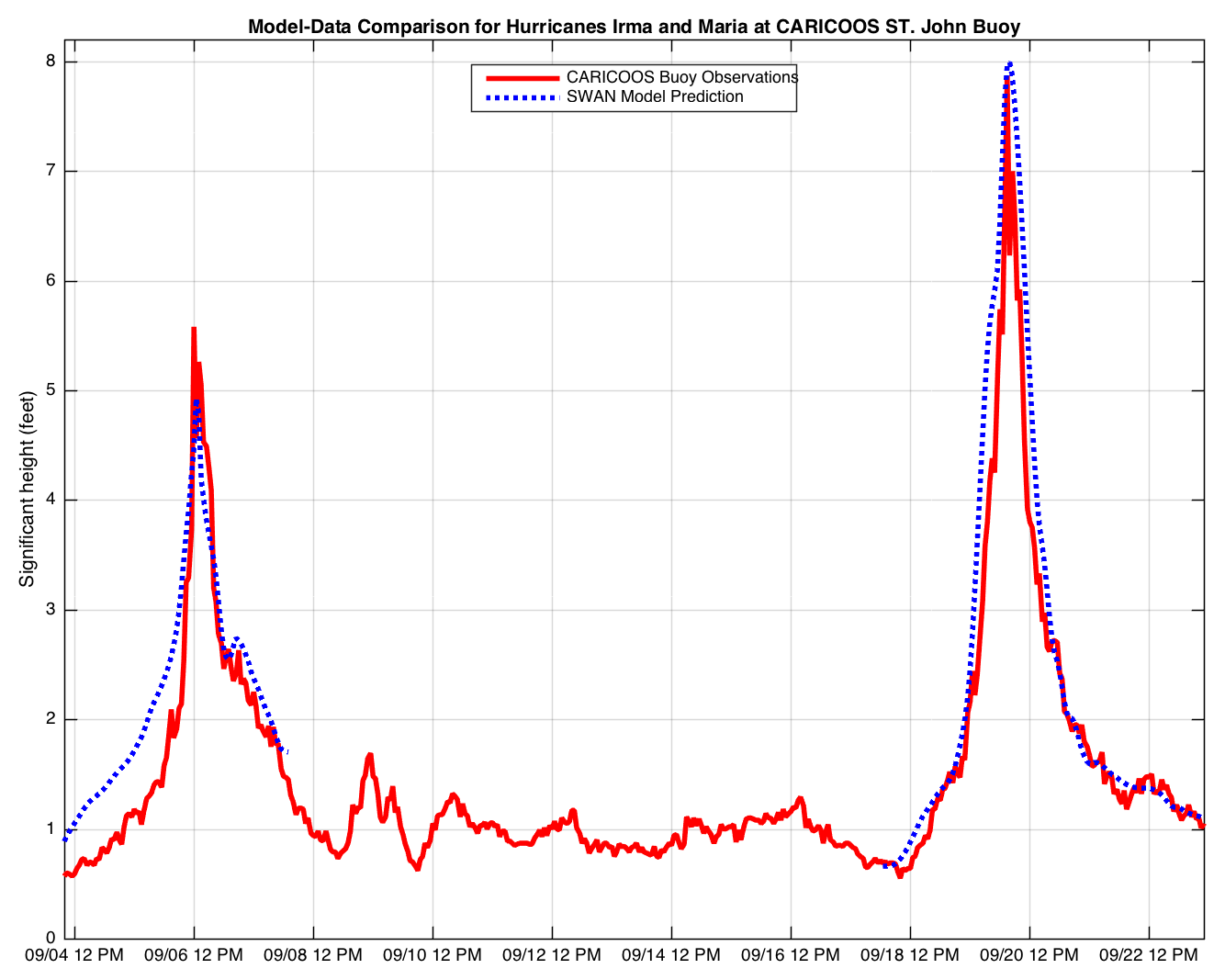


Supplementary Figure S1. Buoy data vs model prediction for significant wave height at the CARICOOS St. John buoy during Hurricanes Irma, Jose, and Maria (See Figure 6 for location). Hurricane Irma hit St. John on 9/6/17, Hurricane Jose passed St. John on 9/9-10/17, while Hurricane Maria passed St. John on 9/20/17.

Supplementary Figure S2 Mean annual sediment production rates from natural and anthropogenic sources on St. John. Bars indicate the range of values.

Supplementary Figure S3. Generalized flowchart of the STJ-EROS model.

**References Cited**

1 Booij, N., Ris, R. C. & Holthuijsen, L. H. A third‐generation wave model for coastal regions: 1. Model description and validation. *Journal of Geophysical Research* **104**, 7649-7666 (1999).

2 team, S. SWAN user manual. (SWAN team, 2012).

3 Anselmi-Molina, C. M. *et al.* Development of an operational nearshore wave forecast system for Puerto Rico and the US Virgin Islands. **28**, 1049-1056 (2012).

4 Canals, M., Morell, J., Corredor, J. E. & Leonardi, S. in *2012 Oceans.* 1-4 (IEEE).

5 Silander, M. F. C. & Moreno, C. G. G. J. R. E. On the spatial distribution of the wave energy resource in Puerto Rico and the United States Virgin Islands. **136**, 442-451 (2019).

6 Taylor, L. A. *et al.* Digital elevation models of Puerto Rico: procedures, data sources and analysis. (2008).

7 Chawla, A. *et al.* A multigrid wave forecasting model: A new paradigm in operational wave forecasting. **28**, 1057-1078 (2013).

8 Zhang, X. *et al.* Representing multiple scales in the Hurricane Weather Research and Forecasting modeling system: Design of multiple sets of movable multilevel nesting and the basin-scale HWRF forecast application. **31**, 2019-2034 (2016).

9 Soulsby, R. & Whitehouse, R. in *Pacific Coasts and Ports' 97: Proceedings of the 13th Australasian Coastal and Ocean Engineering Conference and the 6th Australasian Port and Harbour Conference; Volume 1.* 145 (Centre for Advanced Engineering, University of Canterbury).

10 Jonsson, I. G. in *Proc. 10th Coastal Engineering Conference.* 127-148.

11 Madsen, O. S. in *Proceedings of 24th International Conference on Coastal Engineering.* 384-398 (ASCE).

12 Roulund, A., Sutherland, J., Todd, D. & Sterner, J. in *Scour and Erosion: Proceedings of the 8th International Conference on Scour and Erosion (Oxford, UK, 12-15 September 2016).* 313 (CRC Press).

13 Dixen, M., Hatipoglu, F., Sumer, B. M. & Fredsøe, J. J. C. E. Wave boundary layer over a stone-covered bed. **55**, 1-20 (2008).

14 Deigaard, R. *Mechanics of coastal sediment transport*. Vol. 3 (World scientific publishing company, 1992).

15 Shields, A. Application of similarity principles and turbulence research to bed-load movement. (1936).

16 Dietrich, W. E., Dunne, T., Humphrey, N. F. & Reid, L. M. in *Sediment budgets and routing in forested drainage basins* Vol. 141 5-23 (USDA Forest Service Technical Report PNW-141, 1982).

17 Kondolf, G. M. & Matthews, W. Unmeasured residuals in sediment budgets: a cautionary note. *Water Resources Research* **27**, 2483-2486 (1991).

18 Parsons, A. J. How useful are catchment sediment budgets? *Progress in Physical Geography* **36**, 60-71 (2012).

19 Reid, L. M. & Dunne, T. *Rapid evaluation of sediment budgets*. Vol. 12164 (Catena Verlag Reiskirchen, 1996).

20 Lu, H., Moran, C., Prosser, I. P. & DeRose, R. Investment prioritization based on broadscale spatial budgeting to meet downstream targets for suspended sediment loads. *Water Resources Research* **40** (2004).

21 Walling, D. & Collins, A. The catchment sediment budget as a management tool. *Environmental Science & Policy* **11**, 136-143 (2008).

22 Reid, L. & Dunne, T. Sediment budgets as an organizing framework in fluvial geomorphology. *In: Kondolf, GM; Piégay, H., eds. Tools in Fluvial Geomorphology. Chichester, UK: John Wiley & Sons, Ltd: 357-379. Chapter 16*, 357-379 (2016).

23 Ramos-Scharrón, C. E. & MacDonald, L. H. Development and application of a GIS-based sediment budget model. *Journal of Environmental Management* **84**, 157-172, doi:<http://dx.doi.org/10.1016/j.jenvman.2006.05.019> (2007).

24 Ramos-Scharrón, C. E. *Measuring and predicting erosion and sediment yields on St. John, US Virgin Islands*, PhD Thesis, Colorado State University, (2004).

25 Ramos-Scharrón, C. E. & MacDonald, L. H. Measurement and prediction of natural and anthropogenic sediment sources, St. John, U.S. Virgin Islands. *Catena* **71**, 250-266, doi:10.1016/j.catena.2007.03.009 (2007).

26 Ramos-Scharrón, C. E. & MacDonald, L. H. Measurement and prediction of sediment production from unpaved roads, St John, US Virgin Islands. *Earth Surface Processes and Landforms* **30**, 1283-1304, doi:10.1002/esp.1201 (2005).

27 Swanson, F. J. & Fredriksen, R. L. Sediment routing and budgets: implications for judging impacts of forestry practices. *Sediment budgets and routing in forested drainage basins* **141**, 129-137 (1982).

28 Collins, A. L. & Walling, D. E. Documenting catchment suspended sediment sources: problems, approaches and prospects. *Progress in Physical Geography* **28**, 159-196 (2004).

29 Hoffmann, T. Sediment residence time and connectivity in non-equilibrium and transient geomorphic systems. *Earth-science reviews* **150**, 609-627 (2015).

30 Walling, D. E. The sediment delivery problem. *Journal of hydrology* **65**, 209-237 (1983).

31 De Vente, J. & Poesen, J. Predicting soil erosion and sediment yield at the basin scale: scale issues and semi-quantitative models. *Earth-science reviews* **71**, 95-125 (2005).

32 De Vente, J. *et al.* Predicting soil erosion and sediment yield at regional scales: where do we stand? *Earth-Science Reviews* **127**, 16-29 (2013).

33 Ramos-Scharrón, C. E. & MacDonald, L. H. Runoff and suspended sediment yields from an unpaved road segment, St John, US Virgin Islands. *Hydrological Processes* **21**, 35-50, doi:10.1002/hyp.6175 (2007).

34 Lawler, D. M. The measurement of river bank erosion and lateral channel change: a review. *Earth surface processes and landforms* **18**, 777-821 (1993).

35 Anderson, D. M. *Analysis and modeling of erosion hazards and sediment delivery on St. John, U. S. Virgin Islands*, Colorado State University, (1994).

36 Montgomery, D. R. & Buffington, J. M. Channel-reach morphology in mountain drainage basins. *Geological Society of America Bulletin* **109**, 596-611 (1997).

37 Ramos-Scharrón, C. E. & LaFevor, M. C. Effects of forest roads on runoff initiation in low-order ephemeral streams. *Water Resources Research*, doi:10.1029/2018wr023442 (2018).

38 Ramos-Scharrón, C. E. & LaFevor, M. C. The role of unpaved roads as active source areas of precipitation excess in small watersheds drained by ephemeral streams in the Northeastern Caribbean. *Journal of Hydrology* **533**, 168-179, doi:10.1016/j.jhydrol.2015.11.051 (2016).

39 Dynesius, M. & Jonsson, B. G. Dating uprooted trees: comparison and application of eight methods in a boreal forest. *Canadian Journal of Forest Research* **21**, 655-665 (1991).

40 Luce, C. H. & Black, T. A. Sediment production from forest roads in western Oregon. *Water Resources Research* **35**, 2561-2570 (1999).

41 Robichaud, P. R. & Brown, P. R. *Silt fences: an economical technique for measuring hillslope soil erosion*. (Citeseer, 2002).

42 Gardner, W. H. Water content. *Methods of Soil Analysis: Part 1—Physical and Mineralogical Methods*, 493-544 (1986).

43 Larsen, M. C. Tropical geomorphology and geomorphic work: a study of geomorphic processes and sediment and water budgets in montane humid-tropical forested and developed watersheds, Puerto Rico. (1998).

44 Larsen, M. C., Torres‐Sánchez, A. J. & Concepción, I. M. Slopewash, surface runoff and fine‐litter transport in forest and landslide scars in humid‐tropical steeplands, luquillo experimental forest, Puerto Rico. *Earth Surface Processes and Landforms: The Journal of the British Geomorphological Research Group* **24**, 481-502 (1999).

45 Brooks, G. R., Devine, B., Larson, R. A. & Rood, B. P. Sedimentary development of Coral Bay, St. John, USVI: a shift from natural to anthropogenic influences. *Caribbean Journal of Science* **43**, 226-243 (2007).
